# Understanding how temperature shifts could impact infectious disease

**DOI:** 10.1371/journal.pbio.3000938

**Published:** 2020-11-24

**Authors:** Jason R. Rohr, Jeremy M. Cohen

**Affiliations:** 1 Department of Biological Sciences, Environmental Change Initiative, Eck Institute of Global Health, University of Notre Dame, Notre Dame, Indiana, United States of America; 2 Department of Forest and Wildlife Ecology, University of Wisconsin-Madison, Madison, Wisconsin, United States of America; University of York, UNITED KINGDOM

## Abstract

Climate change is expected to have complex effects on infectious diseases, causing some to increase, others to decrease, and many to shift their distributions. There have been several important advances in understanding the role of climate and climate change on wildlife and human infectious disease dynamics over the past several years. This essay examines 3 major areas of advancement, which include improvements to mechanistic disease models, investigations into the importance of climate variability to disease dynamics, and understanding the consequences of thermal mismatches between host and parasites. Applying the new information derived from these advances to climate–disease models and addressing the pressing knowledge gaps that we identify should improve the capacity to predict how climate change will affect disease risk for both wildlife and humans.

## Abstract 2

Climate change is expected to have complex effects on infectious diseases, causing some to increase, others to decrease, and many to shift their distributions. This Essay explores four major advancements in climate change-disease research and proposes outstanding questions to further advance the field.

## Introduction

**Fig 1:**
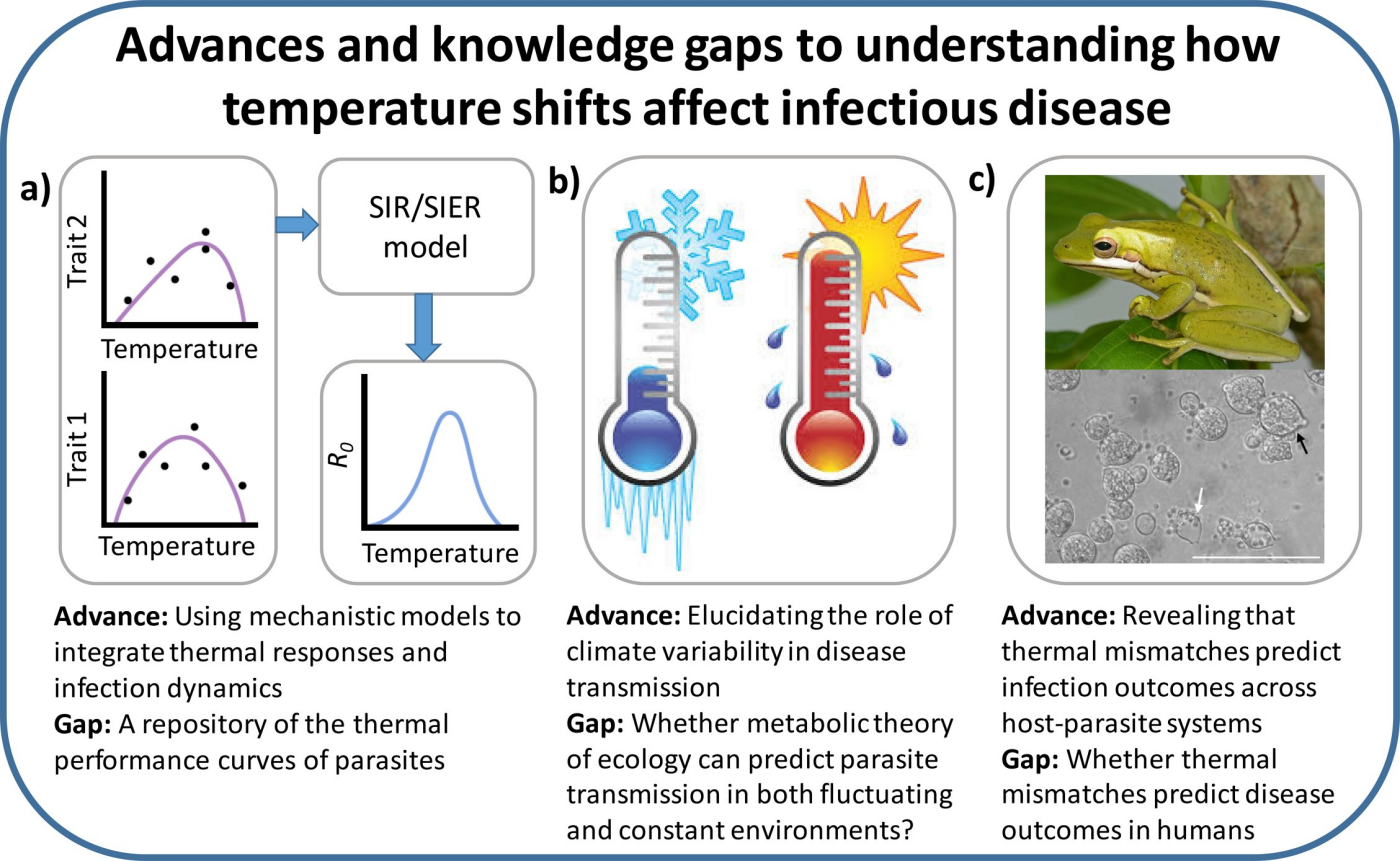
Several important advances to understanding how temperature shifts affect infectious disease have been made in since 2013, when some of the most recent reviews on the topic were published. These advances include **(a)** improvements to mechanistic disease models; **(b)** investigations into the importance of climate variability to disease dynamics; and **(c)** an improved understanding of how thermal mismatches affect host–parasite interactions. Primary knowledge gaps in each of these 3 areas are also provided. SIER, susceptible–exposed–infected–recovered; SIR, susceptible–infected–recovered. *The image in panel b was obtained from a Google Image Creative Commons License Search (**https*:*//webstockreview*.*net/image/cold-clipart-cold-climate/2528297*.*html*?*text=DMCA+Report+-+https%3A%2F%2Fwebstockreview*.*net%2Fimage%2Fcold-clipart-cold-climate%2F2528297*.*html**)*. *The frog image in panel c was provided by Jeremy Cohen*, *and the chytrid fungus image below is available at https*:*//www*.*commons*.*wikimedia*.*org/wiki/File*:*Batrachochytrium_species_morphology_in_culture*.

Temperature and precipitation are often important environmental drivers of infectious disease, including water-borne diseases like cholera [1], vector-transmitted infections like malaria [2], parasitic helminths [3], fungal diseases associated with worldwide amphibian declines [4,5], and marine diseases affecting corals, sea stars, fisheries, and aquaculture [6,7]. Thus, climate change could alter disease dynamics and potentially promote or exacerbate outbreaks in humans and wildlife, inspiring several reviews on climate change’s potential effects [8–12]. These reviews collectively advocated for more empirical work, an assessment of whether diseases would shift, increase, or decrease with climate change, and mechanistic models to move beyond associations toward understanding the drivers of climate change–disease interactions. Several important advances since 2013, when some of the most recent reviews were published, include (1) improvements to mechanistic disease models; (2) investigations into the importance of climate variability to disease dynamics; and (3) an improved understanding of how thermal mismatches affect host–parasite interactions (Fig 1). Here, we discuss these advances and draw on resultant insights to highlight outstanding questions and priorities for further research.

## Using mechanistic models to integrate thermal responses with infection dynamics

### Recent advances

Mathematical models allow researchers to assimilate the multiple underlying processes determining pathogen transmission that are often affected by climate (Fig 1A). Whereas previous modeling approaches assumed linear relationships between temperature and infection dynamics, recent mechanistic models have better captured nonlinear thermal performances of host and parasites [2], which are more realistic because physiological processes decline in performance as temperatures deviate from the optimum, and thus, most traits exhibit a hump-shaped relationship with temperature [13,14]. Indeed, a recent paper discovered that the transmission of 11 different pathogens in 15 mosquito species varied unimodally with temperature [2].

Another value of mechanistic models is their flexibility and extensibility when predicting distributions in novel environments [15]. Mechanistic models are typically parameterized by assimilating experiments testing responses of host and parasite traits to both current and anticipated climatic conditions. In contrast, species distribution models (SDMs) are often correlative and rely strictly on modern or historical weather observations rather than conditions predicted with climate change ([15], but see dynamic SDMs, e.g., [16]). SDMs and mechanistic models performed similarly at predicting current distributions, but predicted differential responses of species to warming [17], prompting some authors to suggest that mechanistic models outperform SDMs in predicting species responses in novel or nonequilibrium contexts, such as with climate change [18].

**Fig 2:**
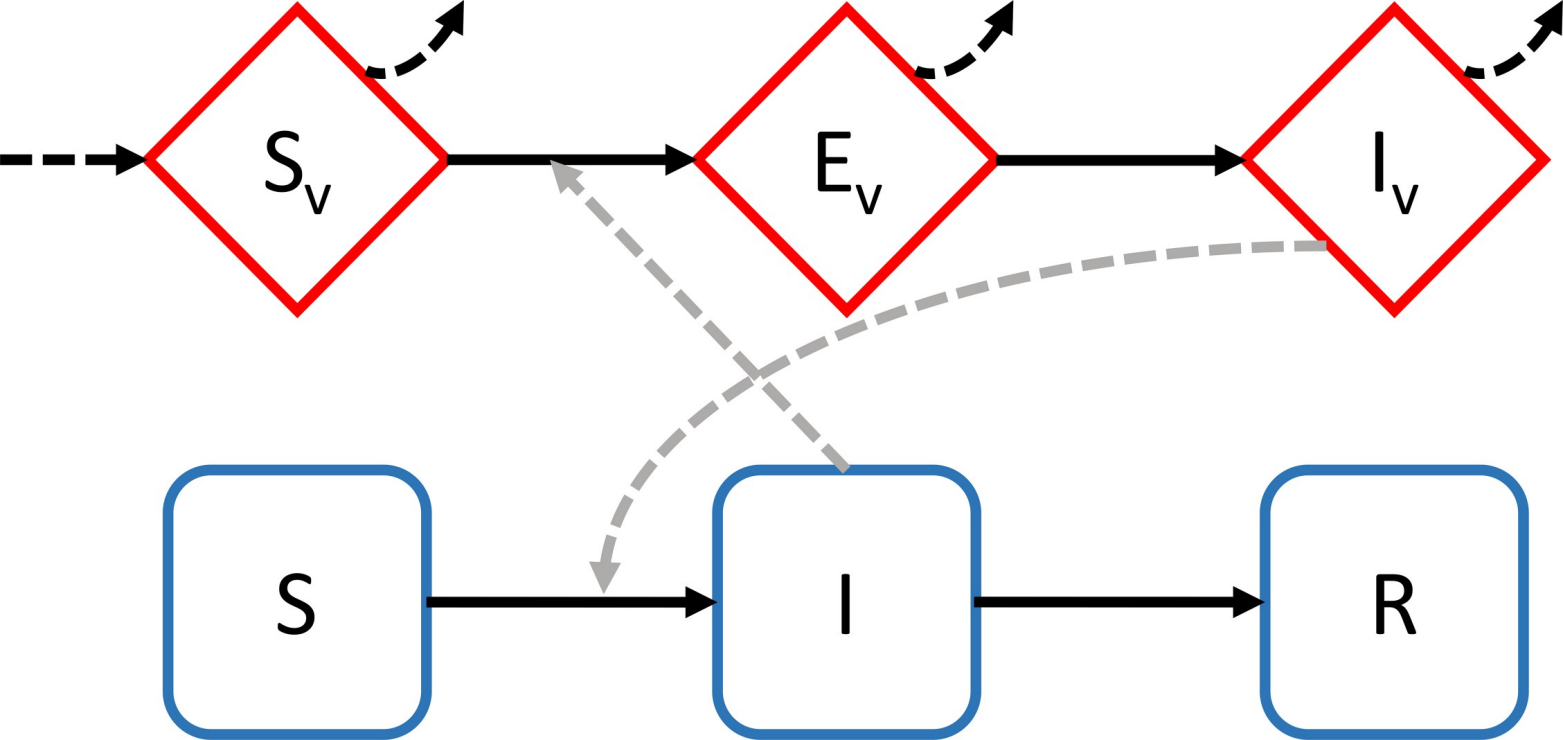
Graphical representation of a simple compartment model for a vector-borne disease. Red diamond compartments represent the vectors and blue squares the host population. S–I–R indicates susceptible, infected, and recovered, respectively, for the hosts. S_v_, E_v_, and I_v_ indicate susceptible, exposed, and infected for the vectors. Solid lines indicate individuals moving between compartments, dashed lines the route of infection, and dotted lines demographic processes in the vectors. We present an example of a vector-borne disease model because it is more complex than models of directly transmitted parasites.

Since 2013, extensions of the mechanistic Ross–MacDonald susceptible–infected–recovered (SIR) transmission model (Fig 2) were used to generate predictions for the transmission of Zika, Chikungunya, and Dengue viruses across temperatures, which were then validated against human case data [19]. In a follow-up work on 11 mosquito-borne pathogens, predictions of peak transmission temperature generated by mechanistic models was up to 6°C lower relative to predictions generated by models with more limited thermal biology assumptions [2]. Accounting for nonlinear thermal traits in mechanistic models also improved predictions for other diseases [20], including dengue [21–23], Ross River virosis [24], and citrus greening [25,26].

Accounting for nonlinearities in thermal traits in mechanistic models also improved predictions for nonvectored parasites of wildlife. For example, Molnar and colleagues [27] generated a mechanistic host–macroparasite model that integrated multiple nonlinear thermal performance curves (TPCs) of the host and parasite to show that climate warming can split a continuous spring-to-fall transmission season of an arctic nematode and its endothermic hosts into 2 separate transmission seasons with altered timings, predictions that closely corresponded to empirical data. Gehman and colleagues [28] quantified the TPCs of several traits of a rhizocephalan parasite and their crab hosts and combined them with field data on seasonal host abundance and parasite prevalence to parameterize a susceptible–exposed–infected transmission model for the purpose of forecasting disease under plausible future climate warming scenarios. Within the coastal southeastern United States, their model predicts that warming will cause sharp declines in parasite prevalence and even local parasite extinction with only 2°C warming, whereas in the northern portion of the parasite’s range, transmission is predicted to increase. These 2 examples, both integrating experiments, field data, and epidemiological models, highlight that the effects of climate change can alter the temporal dynamics of infections and will likely be variable within the geographic range of parasites.

Mechanistic SIR models also have limitations. First, they are data intensive, requiring experiments on multiple traits of the host, vector, pathogen, and their interactions across a range of environmental conditions. Consequently, data are often acquired from experiments with different methodologies and from related species to fill in data gaps, both of which can introduce error [2,19]. Additionally, these models are tailored to specific species, and thus do not easily address questions of generality across host and parasite types (but see [2]).

Advances in mechanistic disease models based on the metabolic theory of ecology (MTE) might help circumvent some limitations of data-intensive, mechanistic SIR models. Several researchers have suggested that thermal dependencies of host and parasite traits (e.g., development and survival) can be described by first principles outlined in MTE [12,29]. If so, then predictions could be generated from well-documented relationships among body size, temperature, and metabolism, potentially reducing the need to quantify relationships between temperature and traits important to parasite transmission and offering null model predictions for data-deficient species [30]. Recently, Kirk and colleagues [29,31] revealed that it is possible to predict the effects of temperature changes on disease from an MTE-based model. This result offers hope that MTE models leveraging well-documented relationships among allometry, temperature, and metabolism can predict effects of climate change on parasite transmission.

### Outstanding questions

Despite recent advances, several outstanding questions exist. First, is it necessary to characterize the thermal dependence of all temperature-dependent traits to predict disease distributions in a changing climate? Addressing this question can be accomplished by comparing the predictions generated by all temperature-dependent traits to those generated by subsets of traits, which would also help to identify the traits that are most influential to disease outcomes. Do MTE-based approaches offer a suitable shortcut for predicting climate–disease associations? Do other shortcuts exist? The application of MTE to infectious disease is nascent, and thus, there is a need to test MTE across host–parasite systems.

Another gap in the literature is a repository of parasite TPCs, despite the existence of similar repositories for thousands of host species [14]. A database of coupled host and parasite TPCs could help resolve which types of parasites might experience the greatest changes in transmission with climate change, where on earth climate change might cause the greatest increases in disease, and how much diseases will expand versus shift their ranges with climate change. Much is also left to be learned about TPCs, such as which components of TPCs (i.e., optima, breadth, or upper or lower limits) have the greatest potential to adapt and acclimate to climate change and whether laboratory-derived TPCs match performances in the field [2,26]. Importantly, practical guidelines have been published recently for the development of experiments and models on the application of both TPCs and MTE to parasitism [30]. These guidelines highlight the importance of capturing the entire thermal response of performance traits, the use of perturbation analyses to determine experimental priorities, experimental design tips for quantifying TPCs, and statistical methods for estimating the parameters of TPCs.

## The role of climate variability in disease transmission

### Recent advances

Two of the hallmarks of climate change are an increase in climate variability and a decrease in diurnal temperature range (DTR), the latter of which is caused by nighttime temperatures warming faster than daytime temperatures [32]. For these reasons, there is interest in the effects of climate variability on disease [5,33–35] and concerns that ignoring variability might compromise climate–disease forecasts [9,11,12]. There are also concerns that increases in unpredictable climatic variation could increase disease because parasites acclimate to temperature shifts faster than hosts, providing windows where parasites could flourish [35,36].

There has been a surge of studies in recent years confirming these concerns (Fig 1B). A series of studies on vector-borne diseases, including malaria [37–39] and dengue [40], demonstrated that, relative to equivalent constant mean temperatures, DTR alters rate processes, such as development, making transmission possible at lower mean temperatures and potentially blocking transmission at higher mean temperatures. Climate variability has also been shown to increase directly transmitted diseases of wildlife, including chytrid fungus, *Batrachochytrium dendrobatidis* (Bd), transmission to amphibians [4,35,41], and a Rickettsiales-like pathogen to abalone [42].

### Outstanding questions

Although variability in temperature can clearly affect pathogen transmission, what is not clear is whether this is because (1) of lags in the adjustment of biochemical and physiological responses of organisms (e.g., acclimation) to temperature shifts [36,43]; (2) TPCs are typically nonlinear and asymmetric; (3) time-averaged rates in fluctuating thermal environments differ from rates in constant environments because of Jensen’s inequality [44,45]; or (4) a combination of these factors. Studies are needed to test how well nonlinear averaging across TPCs estimated at constant temperatures predicts parasite transmission in fluctuating temperature environments [2,34,37]. Additionally, climate change is increasing inter-daily temperature variability while decreasing intra-daily variability, but it remains unclear which timescales of temperature variation are most important, how these scales of variation influence transmission, and how much these patterns depend on parasite type and predictability of the variation [2,35]. Finally, a recent meta-analysis revealed that thermal acclimation rates and thermal breadths are inversely related to body size, consistent with MTE [36]. Hence, future studies should evaluate whether MTE can predict parasite transmission in both fluctuating and constant environments.

## Thermal mismatches between hosts and parasites predict infection outcomes

### Recent advances

**Fig 3:**
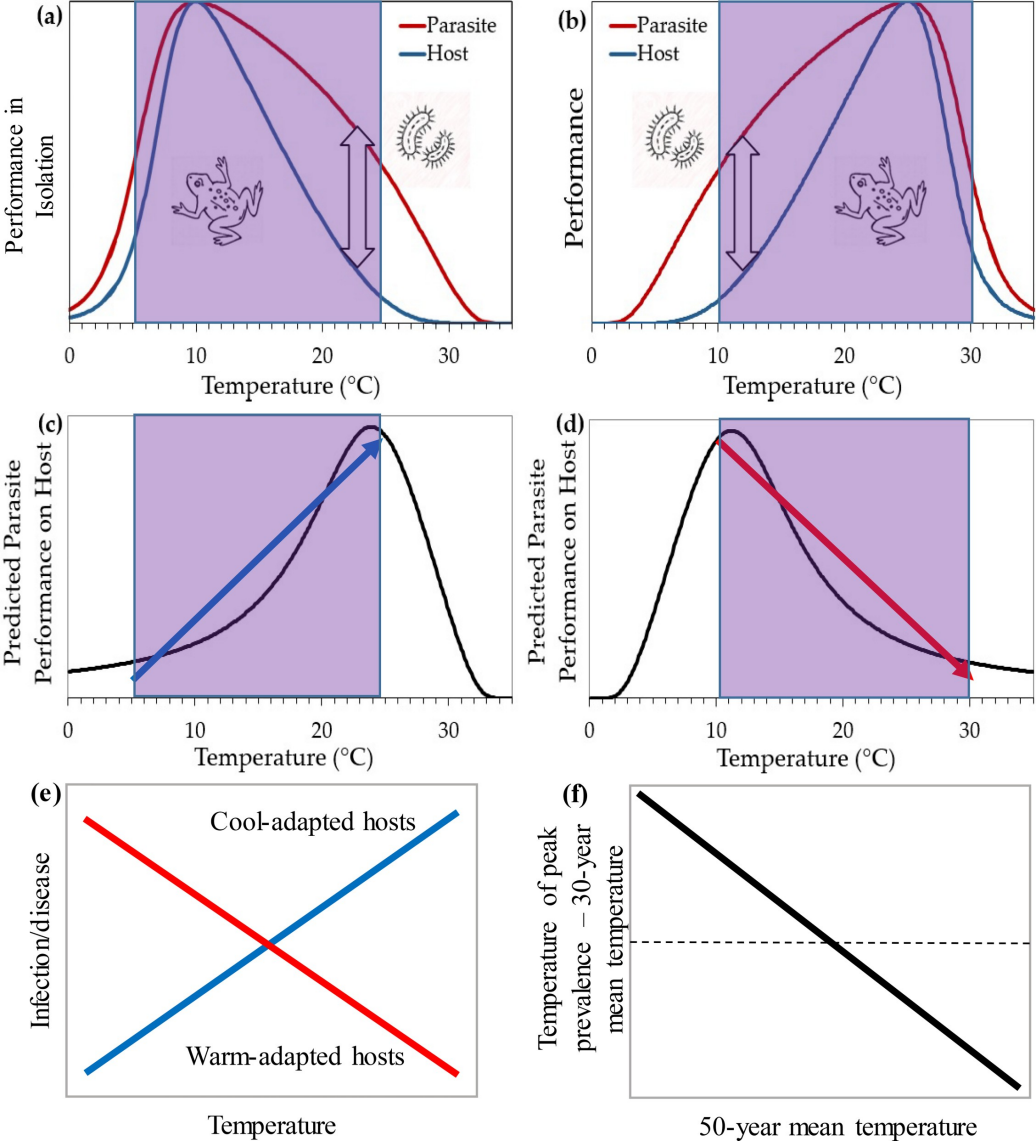
Depiction of the TMH. **(a)** Cold-adapted parasites in isolation most outperform cold-adapted noninfected hosts at warm temperatures (double-sided arrow), whereas **(b)** warm-adapted parasites most outperform warm-adapted hosts at cool temperatures. Hence, when cold- and warm-adapted parasites grow in or on hosts, the TMH predicts that their prevalence will peak at warm **(c)** and cool temperatures **(d)** and be left **(c)** and right skewed **(d)**, respectively. Given that parasites require a minimum, threshold density of hosts to persist [50], the hypothesis assume that there are little to no parasites at temperatures where hosts perform very poorly. Thus, within the threshold performance/density of hosts (purple bands), much of the performance of the parasite growing in or on the host can often be characterized using a linear relationship (blue and red arrows in panels **c** and **d**), despite the relationship being non-monotonic (both increasing and decreasing, i.e., hump shaped) across the entire temperature range. This is because the relationship within this range is predicted to be monotonic and, with error, might often be well fit with a line or some other monotonic function. Given this, the TMH has often been tested 1 of 2 ways: **(e)** by testing for the effect of a statistical interaction between the temperature during disease sampling and the temperature to which host are adapted (usually measured as the 50-year mean temperature at the location of their collection) on infection probability or **(f)** by testing for a negative relationship between the 50-year mean temperature of hosts and the temperature of peak prevalence minus the 50-year mean temperature of hosts. TMH, thermal mismatch hypothesis.

The thermal mismatch hypothesis (TMH), motivated by cases where host and parasite fitness peak at different temperatures under experimental settings [35,46,47], presents a way to explain how changing temperatures impact infection outcomes (Fig 1C). Ultimately, the TMH posits that as environmental conditions shift away from those typically experienced by hosts and parasites (but remain within the threshold density of hosts), parasites often outperform hosts ([4,46,48]; Fig 3). Thus, TMH predicts that parasites reach their highest abundance in hosts in nature at the temperatures they most outperform the host rather than at the temperature they perform best in isolation.

TMH is based on the following well-supported assumptions: Thermal breadths of parasites are greater than hosts [36], and there is local thermal adaptation of hosts and parasites [49] and a threshold density of hosts needed to support parasite populations ([50]; Fig 3A and 3B). Given examples of right-skewed TPCs of cold-adapted species [27,28,31,51,52], possibly driven by cold physiological limits to life, we have only shown both right- and left-skewed TPCs in Fig 3; however, the predictions of TMH have been shown to be independent of the skew of host and parasite TPCs [46]. Based on these assumptions, TMH posits that hosts adapted to cool and warm climates should be at the greatest risk of infection under abnormally warm and cool conditions (thermal mismatches), respectively, because this is where parasites most outperform their hosts (Fig 3A–3F). Thus, hosts from cooler climates should experience higher disease risk with global warming than hosts from warmer climates.

The predictions of the TMH, specifically that cold- and warm-adapted hosts should have peak disease prevalence at relatively warm and cool temperatures, respectively, have been broadly supported using (1) continental- and global-scale analyses of outbreaks of the fungal pathogen Bd across 394 amphibian host species and 1,396 host populations [46,48]; (2) experiments on hosts that can [53] and cannot thermoregulate [46]; and (3) a meta-analysis on host mortality risk from infection across laboratory studies ([54]; Fig 1C). Moreover, TMH better explained the timing and location of >66 declines in the genus *Atelopus* putatively caused by Bd than Bd growth in culture, temperature variability, mean climate alone, climate change alone, or the introduction and spread of Bd [4]. Additionally, in a global analysis, hosts that were larger, from higher elevations and lower latitudes, and of conservation concern were most susceptible to Bd following thermal mismatches, further supporting that thermal mismatches might have contributed to amphibian declines ([48]; Fig 1C).

Recently, support was found for the TMH across 7,346 wildlife populations and 2,021 host–parasite combinations [55]. The strength of support, however, was stronger for ectothermic than endothermic hosts and depended on the pathogen taxon [55]. Projections based on these statistical models and climate change projections suggest that wildlife hosts from temperate and tropical zones will experience sharp increases and moderate reductions in disease risk, respectively, supporting the hypotheses that shifts in infectious disease distributions and net increases in disease globally could occur in the future [55].

Although many researchers have correctly submitted that differences in the TPCs of hosts and parasites can lead to nonlinear host–parasite interactions (i.e., the net outcome of virulence and resistance) as temperatures shifts [28,47], the value of TMH is that it incorporates local adaptation, is derived from first principles of MTE (i.e., the temperature range at which an organism maintains performance is negatively related to body size), and integrates TPCs of the host and parasite but does not require quantification of TPCs for all temperature-dependent traits of each. It can be tested easily by coupling local weather (temperature of peak prevalence) and climate data (30-year mean temperature) with field surveys of infections across temperature, which are plentiful (e.g., [55]). Finally, it offers more nuanced predictions for climate change–disease interactions than past models. It posits that cool-adapted hosts will be at greater risk of disease with climate change than warm-adapted hosts, providing predictions for host populations and species and locations on the planet that might experience the greatest change in disease with a changing climate (e.g., [55]).

Differences in thermal performance of hosts and parasites can also cause mismatches in their phenology [56]. Recent evidence suggests that smaller organisms, such as parasites, phenologically track changes in climate better than larger organisms, such as hosts [56], suggesting that climate change has the potential to phenologically disrupt host–parasite interactions. For instance, the degree of phenological mismatch between hosts and trematodes was a significant positive and negative predictor of behavioral resistance and tolerance of these infections, respectively [57], and climate change–driven disruptions to host–parasite phenology reduced nematode burdens in sheep [58]. Warming of 3°C caused phenological mismatch between hosts and trematodes, halving trematode loads and reducing pathology by 67%, even though total parasite production was similar across temperature treatments [59]. These studies indicate that the degree of phenological synchrony between hosts and parasites can be a driver of the strength and type of host defenses, parasite transmission, disease, and host mortality.

### Outstanding questions

TMH has been tested on hundreds of parasite and wild animal host species and populations [46,48,55], but it has yet to be widely tested on human diseases. Support for TMH is weaker in endothermic than ectothermic hosts [48,55], and humans exhibit numerous disease control measures, and thus, it is unclear whether TMH applies to human diseases. It also remains unclear how well matched the TPCs of hosts and parasites are to their environments and to each other [2]. Additionally, it seems likely that the same first principles that allow parasites to have greater thermal breadths than hosts (e.g., faster rates of acclimation and adaptation, [36]) might also allow parasites to have greater breadths to other components of climate, such as moisture. However, the relative importance of temperature versus other drivers of transmission, such as precipitation, remains understudied. This is especially important given a recent meta-analysis revealing that heavy rainfall and flooding are associated positively with diarrheal diseases [60], and evidence that temperature and moisture can synergistically interact to exacerbate epizootic disease [41].

There are also several outstanding questions regarding phenological mismatches and disease. For example, how important is the level of synchrony in host–parasite phenology relative to total parasite output for predicting climate-driven changes in disease risk? Given that body size is predictive of the strength of phenological shifts [56], can MTE be used as a framework for predicting how climate change might affect synchrony of host–parasite phenology and subsequent disease risk? How frequently does climate change cause host–parasite asynchrony? How long term is any asynchrony given that there should be strong selection for parasites to realign their phenology with that of the host, and their shorter generation times than their hosts should facilitate rapid realignment? Finally, there is also a need to understand the evolutionary responses of host–pathogen interactions to climate change.

## Conclusions

Although there has been considerable progress in advancing understanding of climate change–disease associations, important gaps in the literature remain. These include (1) a repository of parasite TPCs; (2) assessing whether MTE-based approaches offer suitable shortcuts for predicting parasite transmission and host–parasite phenology in fluctuating and constant environments; (3) fully elucidating the evolutionary responses of host–pathogen interactions to climate change; (4) identifying the underlying mechanisms that cause disease to be sensitive to climate variability; (5) determining the timescales of climatic variation that are most important to disease dynamics; and (6) gauging the value of the TMH to predicting human diseases. Addressing these pressing knowledge gaps and applying recent advances to climate–disease models should improve predictions for how changes to climatic means, variances, and extremes will affect disease risk for both wildlife and humans.

**Box 1. A primer on climate-dependent disease transmission models**

Many climate-dependent disease transmission models use an SIR or susceptible–exposed–infected–recovered framework, where coupled differential equations are used to capture transitions to and from each of these compartments (Fig 2). Many variants of these models exist, but most describe the basic reproductive number R_0_, defined as the average number of secondary infections caused by a single infectious individual in a susceptible population. If R_0_ > 1, then the disease is expected to spread, otherwise it will die out.

These models are extended to capture climate by making components of R_0_ depend on extrinsic factors, such as temperature or precipitation. For example, development rate, mortality, and reproduction of hosts, vectors and parasites, host recovery from infection, the biting rate of vectors, and vector competence are often made temperature dependent in dynamical models [2,61,62]. The value of models, when properly parameterized and validated, is that they can integrate the nonlinear effects of climate on each component of R_0_ to generate predictions for how climate change will affect parasite transmission. The vast majority of vector-borne disease models use the Ross–Macdonald formulation of R_0_ and its entomological derivative, vectorial capacity, to study climate–disease dynamics [62]. However, the assumption of homogeneous transmission in a well-mixed population that underpins these Ross–Macdonald-based models is often violated in nature, suggesting that contributions of heterogeneous, focal, climate-related transmission to disease dynamics could be important but remain underexplored [63]. Nevertheless, coupling models with climate and disease data have facilitated groundbreaking discoveries, such as discriminating the effects of intrinsic immunological dynamics from extrinsic climate forcing in cholera [1], revealing that spatiotemporal dynamics of many vector-borne diseases are predictable from the local vector dynamics driven by climatic conditions [61], and determining that the temperature optima for many diseases is lower than previously assumed [2,64,65]. Additional details on modeling infectious diseases can be obtained from the following references: [20,63,66,67].

## Abbreviations

Bd: *Batrachochytrium dendrobatidis*
DTR: diurnal temperature range
MTE: metabolic theory of ecology
SDM: species distribution model
SIR: susceptible–infected–recovered
TMH: thermal mismatch hypothesis
TPC: thermal performance curve
